# The New Hypnotic-Sulphonal

**Published:** 1890-01

**Authors:** 


### The New Hypnotic—Sulphonal.

Sulphonal was first discovered by Prof. E. Baumann, of the University of Freiburg. Its physiological actions and clinical uses were first examined by Professor Kast, who is also a member of the medical faculty at Freiburg University.

Sulphonal has been found to be a reliable hypnotic which has none of the peculiar effects of the narcotics on the nervous and circulatory systems. It has no injurious secondary effects, and may be taken in the proper doses with impunity in order to produce natural, quiet sleep.

It has another great advantage which is of considerable practical importance, viz.: its absolute freedom from taste and odor.

This new hypnotic has been tried quite extensively during the last twelve months, and very favorable results are reported by various observers.

Professor Kast, of Freiburg, published the results of his experiments in the *Berliner Klinische Wochenschrift*, Nov. 16, 1888. He gave the drug to twenty healthy persons, after having convinced himself of its relative harmlessness by experimenting on dogs. These had demonstrated a decided physiological action on the gray cortex of the cerebrum, inducing sound, natural sleep, without any disagreeable after effects. The dose usually employed was 2.0 to 3.0 Gm. (30 to 45 grains). Healthy persons would feel tired and sleepy after its administration, but only a minority of the whole number experimented upon (20) would actually fall asleep and remain asleep for a number of hours. Then sulphonal was given to more than thirty hospital and private patients, and about 120 single observations being recorded. Nervous sleeplessness, due to neurosis or psychoses, insomnia accompanying acute febrile diseases, and sleeplessness of old age were principally selected for the purpose.

Almost without exception sound and quiet sleep was produced within from thirty minutes to two hours after the administration of the drug. No untoward symptoms were observed on awakening, the sleep being as refreshing as if it had been due to natural causes. Pulse, respiration and digestion were not interfered with. The average dose was 30 grains, 15 grains being sufficient for women, while men occasionally required 45 grains.

Another remarkable feature is mentioned by Kast: that no tolerancy is established toward the drug. Its action is the same after many doses have been taken by the same individual as after the first dose.

Similar observations were made by Dr. G. Rabbas, (*Berliner Klinische Wochenschrift*, Nov. 17, 1888): administered sulphonal 220 times to 27 patients suffering from various forms of insanity —*e. g.*, melancholia, mania, general paralysis, etc.

No untoward symptoms are produced, not even when larger and repeated doses are administered—*i. e.*, 60 grains, changing off in short intervals with 30 or 45 grain doses. One patient took one ounce in six consecutive days. Rabbes never saw digestion, respiration or the heart's action unfavorably influenced by sulphonal, and therefore warmly recommends its use.

If given several hours before bedtime sulphonal will produce sound, uninterrupted sleep.

Other observers have noted similar favorable results which have been reported in the various medical journals. The price asked at first deterred many from using it, but since this has been reduced it comes within the reach of all. We predict from an extended experience that this new hypnotic is here to stay.

---

A Good Reason.—Ethel (shuddering)—"How the trees moan and sigh to-night!"

Bobby (speaking whereof he knows)—"Well, I guess you'd moan and sigh if you were as full of green apples as they be."—*Binghamton Republican.*

Dry Operations have been lately advocated by Landerer, of Leipsic, amongst others. His reasons for rendering operations as dry as possible are not without weight. He points out that the employment of all sorts of antiseptics may be dangerous under certain conditions, and that it is advisable to avoid them as much as possible. The employment of sterilized fluids in large quantities is inconvenient and messy, and there are many advantages in keeping the field of operation quite dry. The essential part in the dry method is immediately after the first incision to keep every part of the operation field, not for the moment under the knife, under pressure by means of antiseptic gauze; dry sublimate gauze is what he recommends. By this means the wound can be closed more quickly as the plugging will have arrested the bleeding, and drainage will frequently be avoided altogether. The suturing then takes place, after which a moderately firm compress is applied. In this way the patient is less messed and cooled, there is less loss of blood, and above all, no germs can enter from the moment the plugging is applied. The bleeding is more quickly arrested, and the duration of the operation is shortened, whilst at the same time healing is rendered more rapid and certain.—*Medical Press aud Circular.*

